# Predictors of Unfavourable Outcomes in Children and Adolescents Submitted to Surgical Mitral Valvuloplasty Secondary to Chronic Rheumatic Heart Disease

**DOI:** 10.5935/abc.20190184

**Published:** 2019-10

**Authors:** Renata Cristina Castro Cruz, Bruna Silva Cordeiro, Felipe de Souza Santos, Caroline Rodrigues Fernandes, Julia Maria Alves Gama, Ana Marice Teixeira Ladeia

**Affiliations:** 1 Escola Bahiana de Medicina e Saúde Pública, Salvador, BA - Brazil; 2 Faculdade de Tecnologia e ciências, Salvador, BA - Brazil; 3 Universidade Federal da Bahia, Salvador, BA - Brazil

**Keywords:** Heart Defects,Congenital, Mitral Valve Insufficiency/surgery, Hypertension,Pulmonary, Reoperation, Tricuspid Valve Insufficiency/surgery, Cardiopathy, Rheumatic

## Abstract

**Background:**

Mitral valve repair in paediatric patients with chronic rheumatic heart disease is superior to valve replacement and has been used with good results.

**Objective:**

To identify predictors of unfavourable outcomes in children and adolescents submitted to surgical mitral valvuloplasty secondary to rheumatic heart disease.

**Methods:**

Retrospective study of 54 patients under the age of 16 operated at a tertiary paediatric hospital between March 2011 and January 2017. The predictors of risk for unfavourable outcomes were: age, ejection fraction, degree of mitral insufficiency, degree of pulmonary hypertension, presence of tricuspid insufficiency, left chamber dilation, preoperative functional classification, duration of cardiopulmonary bypass, duration of anoxia, presence of atrial fibrillation, and duration of vasoactive drug use. The outcomes evaluated were: death, congestive heart failure, reoperation, residual mitral regurgitation, residual mitral stenosis, stroke, bleeding and valve replacement. For all analyzes a value of p < 0.05 was established as significant.

**Results:**

Of the patients evaluated, 29 (53.7%) were female, with an average of 10.5 ± 3.2 years. The functional classification of 13 patients (25%) was 4. There was no death in the sample studied. The average duration of extracorporeal circulation was 62.7±17.8 min, and anoxia 50 ± 15.7 min. The duration of use of vasoactive drug in the immediate postoperative period has an average of 1 day (interquartile interval 1-2 days). The logistic regression model was used to evaluate the predictive variables for each unfavourable outcome. The duration of use of vasoactive drug was the only independent predictor for the outcomes studied (p = 0.007). Residual mitral insufficiency was associated with reoperation (p = 0.044), whereas tricuspid insufficiency (p = 0.012) and pulmonary hypertension (p = 0.012) were associated with the presence of unfavourable outcomes.

**Conclusion:**

The duration of vasoactive drug use is an independent predictor for unfavourable outcomes in the immediate and late postoperative period, while residual mitral regurgitation was associated with reoperation, and both tricuspid regurgitation and pulmonary hypertension were associated with unfavourable outcomes.

## Introduction

Chronic rheumatic heart disease (RHD) consists of a non-suppurative complication of rheumatic fever (RF), with uni- or multivalvar involvement, which can lead to severe heart failure.^1^ It is estimated that each year there are 470,000 new cases of RF and 233,000 deaths attributed to RF or RHD.^2^

Mitral valve regurgitation is the main cause of RHD in children;^3,4^ when moderate or severe rheumatic valve disease is associated with pulmonary hypertension and left ventricular dysfunction, the development of congestive heart failure suggests the need for surgical intervention.^3^ Chronic rheumatic disease and its complications generated, in Brazil, 6,648 hospitalizations and a cost of BRL 73,067,919.52 in 2017 alone.^5^

Problems inherent to mitral valve replacement include the need for long-term anticoagulation, risk of bleeding, thromboembolism, endocarditis and lack of growth potential of the prosthesis, which makes the mitral valve plasty (MVP) technique superior to valve replacement in pediatric patients.^6,7^ However, patients submitted to valvuloplasty had a higher reoperation rate in the short term.^8^

This study aimed to identify predictors of unfavorable outcome in children and adolescents submitted to mitral valvuloplasty secondary to rheumatic heart disease.

## Methods

A retrospective cohort study was performed. Data were collected by reviewing information on medical records (physical and electronic). The collection was performed by four researchers after standardized training. The Escola Bahiana de Medicina e Saúde Pública Research Ethics Committee approved this study together with CAAE from 64019316.0.0000.5544.

### Population

The study included 54 patients with mitral insufficiency of rheumatic etiology who underwent surgical correction by MVP technique, from March 2011 to January 2017.

### Preoperative evaluation

Patients were clinically identified using the New York Heart Association (NYHA) Functional Classification.^9^ All medications that patients used continuously for at least one month were recorded. Valvular lesions were assessed by preoperative transthoracic echocardiography, classifying the lesions as “absent/discrete” or “moderate/significant”. Patients who presented another cause of valve damage at the time of surgical correction by MVP (infective endocarditis; congenital, post-traumatic, degenerative lesions or dystrophic lesions; cardiomyopathies or inflammatory or ischemic disease) or who underwent aortic valve surgery or other procedures in the same surgical time of MVP or an undocumented previous MVP, or patients who did not reach 60 postoperative days until January 2017 were excluded from the study.

### Surgical technique

The reconstructive valve surgery technique was MVP, described by Carpentier,^10^ which includes annuloplasty and commissurotomy. The patients studied were preferably operated by the same medical team. The intraoperative data collected were: surgical technique used, duration of cardiopulmonary bypass (CPB), duration of anoxia and presence of atrial fibrillation. The intraoperative outcomes studied were: arrhythmia, cardiorespiratory arrest (CRA) and bleeding.

### Follow-up

Follow-up was carried out within 60 days after surgery, in an outpatient setting, in a single centre. The predictors of risk for unfavourable outcomes studied were: age, ejection fraction, type of valve lesion, degree of mitral insufficiency (MI), left chamber dilatation, NYHA Preoperative Functional Classification, surgical technique used, duration of CPB, duration of anoxia, presence of atrial fibrillation, presence of pulmonary hypertension (PH) (sPAP > 35 mmHg) and presence of tricuspid insufficiency (TI).

Early (up to 7 postoperative days) and late (>7 days post-operative) outcomes related to heart valve disease were studied. The following were investigated: death, heart failure, cardiogenic shock, endocarditis, mitral valve damage, sepsis, stroke, bleeding, reoperation and valve replacement. The presence of any of these outcomes alone or in combination would characterize an unfavourable outcome as a single dependent variable.

### Statistical analysis

The Statistical Package for Social Sciences (SPSS Inc., Chicago, IL, USA), version 14.0 for Windows, was used for the elaboration of the database and for descriptive analysis. The results were presented in tables. Categorical variables were expressed in frequencies and percentages. Continuous variables with normal distribution were expressed as mean and standard deviation; those with non-normal distribution were expressed in median and interquartile range. The normality of the numerical variables was verified through descriptive statistics, graphical analysis and the Kolmogorov-Smirnov test.

The independent Student’s *t* test was used to compare groups of numerical variables with normal distribution (age, weight, body mass index - BMI, duration of anoxia, duration of CPB, ejection fraction). The Mann-Whitney test was used to compare numerical variables with asymmetric distribution, such as duration of use of vasoactive drugs (VAD).

The χ^2^ test was used to compare the use of medications in the preoperative and upon hospital discharge, and the intergroup comparison of the following categorical variables: gender, origin, outpatient follow-up, reoperations, functional classification, surgical team, events during surgery, duration of extubation and echocardiographic variables. When the distribution showed n < 5 individuals in each category, Fisher’s exact test was used.

The paired Student’s t-test was used for the numerical variable “ejection fraction” in the comparison of the paired groups (pre- and postoperative), and the McNemar test was used to compare the categorical variables of the echocardiogram. For all univariate analyses, a value of p < 0.05 was established.

The logistic regression model was used to evaluate the predictive variables for unfavourable outcomes in children and adolescents who underwent surgical mitral valvuloplasty secondary to rheumatic heart disease. After the univariate analysis, the independent variables were included in the logistic model if they presented p < 0.05, remaining in the model if they remained significant (p < 0.05). The manual procedure for insertion and withdrawal of the variables was adopted. Results were presented using Odds Ratio (OR) and their respective 95% confidence intervals (95%CI).

## Results

From March 2011 to January 2017, 90 patients underwent surgery by the MVP technique in the tertiary hospital where the present study was carried out. Of these, 36 were excluded, of which 7 had congenital mitral lesions (mitral dysplasia), 8 had underwent aortic valve replacement associated with MVP and 21 due to loss to follow-up or incomplete data.

### Characteristics of the sample studied

Table 1 shows clinical and demographic aspects of the 54 patients included, of which 29 (53.7%) were female, with a mean age of 10.5 ± 3.2 years. Of these patients, 34 (64.2%) lived on the countryside of the State of Bahia, 5 (9.4%) were from the Metropolitan Region and 14 (26.4%) were from the capital, Salvador. The mean BMI was 15.7 ± 3.5 kg/m^2^. Prior to the surgery, 27 (51.9%) had regular outpatient follow-up. The disease duration until the surgery was a median of 8 months (interquartile range 5-36). The functional classification of 44 (81.48%) patients was between NYHA 2 and 4, with 13 (25%) being NYHA 4. None presented atrial fibrillation or had to undergo emergency surgery. There were no deaths in the sample studied.

**Table 1:** Characterization of sociodemographic and clinical variables of 54 children and adolescents with rheumatic mitral insufficiency undergoing mitral valvuloplasty

Variables	Mean ± SD
Age (years)	10.5 ± 3.2
Weight (kg)	32.9 ± 14.3
Height (m)	1.4 ± 0.2
Body mass index (BMI) (kg/m^2^)	15.7 ± 3.5
	**Median (IQ25–IQ75)**
Time from disease to surgery (months)	8.00 (5.00–36.00)
Sex	n (%)
Female	29 (53.7)
Male	25 (46.3)
**Origin**	
Countryside	34 (64.2)
Salvador	14 (26.4)
Metropolitan Region	05 (9.4)
**Outpatient follow-up**	
Regular	27 (51.9)
Irregular	25 (48.1)
**Cardiac insufficiency**	
NYHA 1	10 (18.5)
NYHA 2	22 (40.7)
NYHA 3	9 (16.7)
NYHA 4	13 (24.1)

SD: standard deviation; NYHA: New York Heart Association.

Only 3 (5.6%) patients had to undergo reoperation, all of them undergoing a single reoperation. The causes were moderate to severe aortic regurgitation, leading to valve replacement in one patient on the 23^rd^ postoperative day, and the other on the 45^th^. The third patient maintained severe MI even after correction, evolving with associated severe aortic insufficiency, undergoing reoperation for aortic and mitral valve replacement on the 45^th^ day after repair.

Preoperative medications were grouped into 4 combinations: combination 1 - captopril and furosemide; combination 2 - captopril, furosemide and spironolactone; combination 3 - captopril, furosemide, spironolactone and digoxin; combination 4 - captopril, furosemide, spironolactone and carvedilol. Of these combinations, approximately half of the patients (55.8%) used combination 1. All patients had regular use of benzathine penicillin.

### Intraoperative data

The patients underwent surgery with a standard surgical team in most cases, with only 2 procedures (3.8%) being performed by another team. The most used surgical technique was annuloplasty (96.2%), followed by commissurotomy 02 (3.8%), considering only the main procedure. There were events during surgery in 24 (44.4%) of the cases, including: severe bleeding (2), CRA (6), use of VAD (8) or others (8). The mean duration of CPB was 62.7 ± 17.8 min and anoxia was 50 ± 15.7 min. Extubation occurred within 6 hours postoperatively in 48 (92.3%) patients. The duration of VAD use in the immediate postoperative period had a median of 1 day (interquartile interval 1-2 days).

### Description of preoperative and postoperative echocardiograms

Table 2 describes the data found in the preoperative and immediate postoperative echocardiogram (up to 7 postoperative days), comparing their results. The postoperative ejection fraction was reduced when compared to the preoperative one, 54.8 ± 13.9% and 70.2 ± 8.5%, respectively, with a value of p < 0.05. As for left chamber dilatation, 98% of the patients presented it preoperative and 87% postoperatively. This reduction showed p = 0.063, demonstrating a trend towards significance.

**Table 2:** Description of surgical and echocardiographic variables in the preoperative and immediate postoperative periods of 54 children and adolescents with rheumatic mitral insufficiency undergoing mitral valvuloplasty

Variables		Preoperative Mean ± SD	Post-operative Mean ± SD	p value
Duration of ECC (min)		62.7 ± 17.8	---	---
Duration of anoxia (min)		50.0 ± 15.7	---	---
Duration of VAD use (days)		---	1.0 (1.0 - 2.0)	---
Ejection fraction (%)		70.2 ± 8.5	54.8 ± 13.9	0.015^§^
**Dilation of left chambers**		**n (%)**		
No		01 (2.0)	06 (11.1)	0.063^¥^
Yes		48 (98.0)	32 (84.2)	
**Mitral stenosis**				
Absent/discrete		46 (93.9)	36 (94.7)	1.000^¥^
Moderate/significant		03 (6.1)	02 (5.3)	
**Aortic insufficiency**				
Absent/discrete		36 (73.5)	30 (78.9)	1.000^¥^
Moderate/significant		13 (26.5)	08 (21.1)	
**Aortic stenosis**				
Absent/discrete		49 (100.0)	38 (100.0)	---
**Mitral insufficiency**				
Absent/discrete		01 (2.0)	32 (84.2)	0.000^¥^
Moderate/significant		48 (98.0)	06 (15.8)	
**Tricuspid insufficiency**				
Absent/discrete		36 (73.5)	31 (81.6)	0.508^¥^
Moderate/significant		13 (26.5)	07 (18.4)	
**Pulmonary hypertension**				
No		11 (22.4)	31 (81.6)	0.000^¥^
Yes		38 (77.6)	07 (18.4)	

ECC: extracorporeal circulation; VAD: vasoactive drug;

§ paired Student’s t test;

¥ McNemar test.

Among the valve changes described in Table 2, moderate or significant aortic insufficiency was present in 13 (26.5%) patients in the preoperative period. In the postoperative period, only 8 (21.1%) had aortic insufficiency, but with no statistical significance (p = 1,000).

Moderate or significant MI was present in 48 (98%) patients in the preoperative period. Of these, none of the patients’ MI had worsened and 6 (15.8%) patients maintained moderate or significant postoperative MI (p < 0.001).

PH was present in 38 (77.6%) patients in the preoperative period and only 7 (18.4%) in the postoperative period. No patient progressed to PH or worsened in the postoperative period. On the other hand, 31 (81.6%) participants who had preoperative PH did not present it in the postoperative period (p < 0.001).

### Analysis and description of outcomes

The presence of outcomes in the sample was divided into outcomes in the immediate postoperative period (up to 7 days) and in the late postoperative period (up to 60 days), as shown in Table 3. Seventeen patients presented an immediate postoperative outcome, mitral lesion (stenosis and/or residual insufficiency), being present in 8 (14.8%) patients. In the late postoperative period, 16 had outcomes, and mitral regurgitation was again the most common, presented by 11 (20.4%) patients.

**Table 3:** Description of the immediate and late outcomes (and combined outcomes) of 54 children and adolescents with rheumatic mitral disease undergoing mitral valvuloplasty

Variables		n (%)
**Immediate outcome (n = 54)**		
None		37 (68.6)
CHF		01 (1.9)
Sepsis		01 (1.9)
Mitral lesion*		04 (7.4)
Others		04 (7.4)
Bleeding and others		02 (3.7)
CHF and mitral lesion *		01 (1.9)
Others and CHF		01 (1.9)
Mitral lesion* and others		02 (3.7)
Mitral lesion* and bleeding		01 (1.9)
**Late outcome (n = 54)**		
None		38 (70.4)
CHF		01 (1.9)
Reoperation/valve replacement (up to 30 days)		03 (5.6)
Mitral lesion*		11 (20.4)
Others		01 (1.9)

* mitral lesion: mitral stenosis and/or residual mitral insufficiency;

n: number of participants; CHF: congestive heart failure.

The comparison between the use of medications in the preoperative period and after discharge and the outcomes did not present statistical significance, regardless of the combination used.

The variables that were related to the presence of late postoperative outcome were duration of CPB and duration of anoxia, both with p < 0.05. Age, weight, height, gender, origin, outpatient follow-up, disease duration until surgery, functional classification, surgical team, surgery events and duration of extubation had no statistically proven relationship.

When the variables of the echocardiogram were studied in the immediate and late postoperative period, as shown in Table 4, the relationship between the presence of outcomes with MI, TI, and PH is statistically significant. In preoperative echocardiography, no relationship of statistical significance with the outcomes was found.

**Table 4:** Comparison between clinical and echocardiographic variables in the postoperative period with clinical outcomes in 54 children and adolescents with rheumatic mitral insufficiency undergoing mitral valvuloplasty

Variables	Imediate postoperative period (up to 7 days)		p value	Late postoperative period (up to 60 days)		p value
	Yes mean ± SD	No mean ± SD		Yes mean ± SD	No mean ± SD	
Duration of VAD use (days)	3.0 (1.0–3.0)	1.0 (1.0–2.0)	0.009^¶^	2.0 (1.0–3.0)	1.0 (1.0–2.0)	0.035^¶^
Ejection fraction (%)	54.7 ± 17.6	54.9 ± 10.6	0.982*	54.5 ± 15.8	55.0 ± 13.0	0.936*
**Dilation of left chambers**	**n (%)**	**n (%)**		**n (%)**	**n (%)**	
No	02 (14.3)	04 (16.7)	0.846^¥^	02 (16.7)	04 (15.4)	0.920^¥^
Yes	12 (85.7)	20 (83.3)		10 (83.3)	22 (84.6)	
**Mitral stenosis**						
Absent/discrete	12 (85.7)	24 (100.0)	0.057^¥^	10 (83.3)	26 (100.0)	0.094^¥^
Moderate/significant	02 (14.3)	00 (00.0)		02 (16.7)	00 (00.0)	
**Aortic insufficiency**						
Absent/discrete	10 (71.4)	20 (83.3)	0.385^¥^	09 (75.0)	21 (80.8)	0.685^¥^
Moderate/significant	04 (28.6)	04 (16.7)		03 (25.0)	05 (19.2)	
**Aortic stenosis**						
Absent/discrete	14 (100.0)	24 (100.0)	---	12 (100.0)	26 (100.0)	---
**Mitral insufficiency**						
Absent/discrete	09 (64.3)	23 (95.8)	0.010^¥^	08 (66.7)	24 (92.3)	0.044^¥^
Moderate/significant	05 (35.7)	01 (4.2)		04 (33.3)	02 (7.7)	
**Tricuspid insufficiency**						
Absent/discrete	09 (64.3)	22 (91.7)	0.036^¥^	07 (58.3)	24 (92.3)	0.012^¥^
Moderate/significant	05 (35.7)	02 (8.3)		05 (41.7)	02 (7.7)	
**Pulmonary hypertension**						
No	09 (64.3)	22 (91.7)	0.036^¥^	07 (58.3)	24 (92.3)	0.012^¥^
Yes	05 (35.7)	02 (8.3)		05 (41.7)	02 (7.7)	

n: number of participants; SD: standard deviation; IQ: interquartile range; VAD: vasoactive drugs;

* independent Student’s t test;

¶ Mann-Whitney’s test;

¥ χ^2^ test or Fisher’s exact test.

### Predictive variables

The predictive variables are presented in Table 5. In the final model, the variable duration of VAD use was found as an independent predictor for the immediate outcome, presenting OR 2.5 (95%CI, 1.3-4.9), while no independent predictor was found for the late outcome. The duration of ECC was close to statistical significance.

**Table 5:** Predictive variables for immediate and late outcomes in 54 children and adolescents with rheumatic mitral insufficiency undergoing mitral valvuloplasty

Variables		Input Model		Final Model	
		OR (95%CI)	p value	OR (95%CI)	p value
**Immediate outcome**					
Sex		3.6 (0.8–15.0)	0.084	-	-
Duration of VAD use (days)		2.4 (1.2–4.9)	0.014	2.5 (1.3–4.9)	0.007
**Late outcome**					
Days of VAD		1.8 (0.9–3.7)	0.95	-	-
Number of reoperations		1.8 (0.1–31.7)	0.683	-	-
Duration of ECC (min)		1.0 (0.9–1.1)	0.538	1.0 (1.0–1.1)	0.051
Duration of anoxia (min)		1.0 (0.9–1.1)	0.958	-	-
Extubation (hour)		5.2 (0.4–67.3)	0.211	-	-

OR: Odds Ratio; 95%CI: 95% confidence interval; logistic regression.

## Discussion

MVP is universally accepted as superior to valve replacement (bioprostheses or metal prostheses), especially in children in whom growth, problems with anticoagulation, thromboembolism, rapid valve degeneration, increased risk of endocarditis and less preservation of ventricular function are unfavourable factors to this technique.^6-8,10-12^ In the tertiary hospital in which the study was performed, MVP is the preferred technique.

Patients in the study were followed up for two months after MVP, and in that period, there were no deaths in the sample studied. This is in accordance with the literature, in which the precocious or hospital mortality rate ranged from 0.9 to 3.5%.^7,12,13^

The literature lacks in studies that identify probable clinical predictors of negative outcomes in patients undergoing MVP surgery. In the present study, PH presented statistical significance for both immediate (≤7 days) and late (up to 60 days) postoperative outcomes in the univariate analysis, but did not maintain significance in the multivariate analysis. In their study of 122 mitral repairs, Kim et al.,^14^ after univariate and multivariate analysis, found preoperative pulmonary hypertension as the only independent risk factor for death (HR: 3.75 95%CI: 1.21-11.57; p = 0.022), but this study was carried out with adults with a mean age of 48.9 ± 11.5 years. The results in this study may differ from those found in the literature due to the sample size, as well as the age: the younger the sample, the higher the mortality rate and the higher the early rate of valve failure.^15-17^

In addition, postoperative TI was also a predictor of univariate analysis in this study, both in the immediate postoperative period and in the late postoperative period, but it lost significance in the multivariate analysis. No associations were found in the literature reviewed, but, like PH, TI is a criterion that should be valued. Although they were not independent predictors of outcomes, both variables were associated with unfavourable outcomes, both in the immediate and late postoperative period, with statistical significance, and should be used as initial points for surgical indication and follow-up in these patients.

Yakub et al.^13^ described residual mitral regurgitation ≥2 crosses as a predictor of valve failure and reoperation. In our study, the presence of moderate or significant residual MI in the postoperative period was associated with outcome both in the immediate and late postoperative period, in the univariate analysis, with statistical significance. Of the three children who underwent valve replacement, one presented moderate to severe mitral regurgitation even after attempted repair by MVP technique and the other two developed moderate to severe aortic regurgitation and underwent aortic valve replacement before completing sixty postoperative days. This data is in agreement with the literature in other studies as well, such as in those by Silva et al.^11^ and Severino et al.,^15^ always being a marker for reoperation. It is known that the late MVP results also depend on good coaptation of the cusps, which can be obtained through association of surgical techniques and reassessed at the end of the repair, whenever possible with intraoperative transesophageal echocardiography,^18^ which is not available in the hospital in which the study was carried out.

The mean duration of ECC found was 62.7 ± 18.8 minutes and the duration of anoxia was 50.0 ± 15.7 minutes, showing an association with the outcomes in the immediate postoperative period. In fact, in the literature, duration of CPB has been described as an independent predictor for cardiac surgeries, usually due to inflammatory factors in the bloodstream. Thus, it is well established that a CPB time longer than 90 minutes is associated with a more complicated postoperative period. The study by Talwar et al.^16^ showed duration of CPB of 47.6 ± 11.9 and duration of anoxia of 37.2 ± 12.8, and when assessed for association with early death or reoperation, no significance was found.^16^ This divergence with the literature may be due to the longer duration of both CPB and anoxia found in our sample.

The ECC time found in this study may have been influenced by variables that were not studied, such as anatomical differences, anterior leaflet involvement, thickening of the ribs, calcifications and papillary muscle involvement. Non-standardization of intraoperative and echocardiographic records, as well as the retrospective nature of the study, prevented the analysis of these data.

The duration of VAD use in the immediate postoperative period was found as a predictor for outcome in this study. Silva et al.^11^ also described the use of VAD, finding OR of 1.47 (95% CI 0.32-6.83), but they did not present statistical association in their sample. Other variables may have influenced this divergence in results, such as longer duration of CPB and anoxia in the study by Silva et al.^11^

The use of drugs for clinical optimization of CHF, due to the volume overload generated by mitral annular dilatation, also did not present significance and no description of the study of this variable was found in the reviewed literature, except in the study by Silva et al.,^11^ in which there is only a description of the use of anticongestive medications in 40% of patients in the preoperative period, not presenting a statistical analysis of this data.

The variables age, weight, height, duration of illness until surgery, NYHA preoperative classification, presence of atrial fibrillation and use of drugs were not significant in this study. In the studies by Talwar et al.^16^ and Kalfa et al.,^17^ these data were evaluated descriptively without statistical analysis and using only residual MI, reoperation, valve replacement and mortality as the outcome, without considering other variables such as bleeding, CHF and sepsis as possible outcomes.

The literature presents other outcome predictors that were not identified or not studied in our sample, such as ventricular dysfunction, studied by Talwar et al.,^16^ with HR 4,9 (95%CI 2.65-9.2), p < 0.005. On the other hand, Yakub et al.^13^ described the NYHA preoperative classification, emergency surgeries and double valvular lesions as predictors of early death.

In our country, rheumatic disease is the main cause of acquired heart diseases in childhood and adolescence, unlike in developed countries, where Kawasaki disease is the most frequent cause of acquired heart disease in the paediatric age group.^19,20^ In this context, degenerative lesions are the main indications of mitral valve repair, which justifies the small number of studies found, taking into account only valvular corrections by MVP technique for valve sequela due to chronic RHD in the pediatric age group.^21^

Although Brazil is considered a high risk country for RF, with 40% of heart surgeries being performed for valve repairs due to chronic RHD sequelae, according to data from the Department of Informatics of the Unified Health System (DATASUS),^5^ only a few Brazilian studies were found involving the theme addressed in the present study, and only one of them comprising the paediatric age group, all focusing on surgical results. Murad et al.^7^ studied 86 patients with a mean age of 35.8 years and concluded that MVP can be performed with low mortality and should be the procedure of choice in patients with MI. Similarly, Pomerantzeff et al.^22^ studied 330 patients with a mean age of 26.9 ± 15.4 years for 20 years and concluded that the MVP technique is feasible in rheumatic patients with low early and late mortality. Severino et al.,^15^ in a study with 104 adult patients (mean age 32.73 ± 14.74 years) evaluating MVP results in rheumatic patients, found that late reoperation was associated with postoperative residual MI (p < 0.001), presence of PH (p < 0.01), age (p < 0.04) and postoperative functional classification (p < 0.001). Silva et al.^11^ evaluated the outcome of valve reconstruction in rheumatic lesions in 40 patients younger than 18 years after 4 years of evolution and did not find statistically significant risk factors that could interfere with the evolution of patients in relation to valve replacement before 4 years. The studied variables included: functional classification in the pre- and postoperative period, amount of drugs used by patients at the time of surgery, duration of CPB and anoxia and need for VAD in the immediate postoperative period.

To date, no study has been published in the North and Northeast Regions involving patients with chronic RHD, although the socioeconomic characteristics of these regions are strong factors for a higher prevalence of chronic RHD in the country. In the Brazilian articles published by Severino et al.^15^ and by Silva et al.^11^ there is no report of the origin of the patients operated by the MVP technique, nor were they intended to look for possible predictors for other unfavourable outcomes besides reoperation and mortality.

Because the surgical indications for mitral repair in the paediatric population do not have well-defined criteria in the literature, they require previous discussions and interaction between paediatric cardiologists, cardiologists and cardiac surgeons in order to delineate and minimize factors that may contribute to a postoperative period in the short, medium and long term with good results.

This is a pioneer study, due to the characteristics already described, in an exclusively paediatric population, in a high-risk region for chronic RHD, which should elicit new discussions regarding the topic addressed. Because this pathology has the special characteristic of being one of the rare rheumatic diseases whose etiologic agent is known and, therefore, has a specific treatment and can be avoided with the adoption of low-cost preventive measures with high effectiveness. Being the primary prophylaxis performed with low-cost and easily accessible antibiotics, valve sequelae and, consequently, cardiac surgeries could be avoided, reducing cost to society and improving the quality of life for the affected population.

## Conclusion

The time of VAD use was an independent predictor for outcomes studied postoperatively. Residual MI was associated with reoperation, while TI and PH were associated with unfavourable outcomes in the immediate and late postoperative period. The data in this study allow new investigations to improve the prognosis of children and adolescents with chronic RHC submitted to the MVP repair technique.

### Study limitations

The limitations of our study are related to its retrospective nature, sample size, and due to it being carried out in a single centre.
